# Data on molecular characterization and gene expression analysis of secretory carrier-associated membrane protein 5 (SCAMP5) from the red sea bream (*Pagrus major*)

**DOI:** 10.1016/j.dib.2019.103901

**Published:** 2019-04-04

**Authors:** Min Jin Heo, Andre Kim, Chan-Il Park

**Affiliations:** a Institute of Marine Industry, College of Marine Science, Gyeongsang National University, 455, Tongyeong 650-160, Republic of Korea; b Department of Pharmaceutical Engineering, College of Medical and Life Science, Silla University, Busan 617-736, South Korea

**Keywords:** Secretory carrier membrane protein 5, Red sea bream, *Edwardsiella piscicida*, *Streptococcus iniae*, Red sea bream iridovirus

## Abstract

Secretory carrier membrane proteins (SCAMPs) are widely distributed integral membrane proteins implicated in membrane trafficking. Secretory carrier membrane protein 5 (SCAMP5) is expected to be involved in regulation of the immune response because it is expressed in a variety of immune tissues and promotes the secretion of cytokines in monocytes and macrophages. In this study, we performed an analysis of the molecular characteristics and phylogenetic of the SCAMP5 gene identified in *Pagrus major* (PmSCAMP5). In addition, we analysed PmSCAMP5 gene expression levels in the tissues of red sea bream infected with various pathogens [*Edwardsiella piscicida* (*E. piscicida*), *Streptococcus iniae* (*S. iniae*) and Red sea bream iridovirus (RSIV)], and we analysed PmSCAMP5 gene expression levels in the tissues of healthy red sea bream. This study was carried out to provide basic data on the non-specific immune system of the red sea bream.

**Table:** Specifications table

Subject area	*Immunology and Microbiology*
More speciﬁc subject area	*Immunology*
Type of data	*figure*
How data was acquired	*Data were collected using Real-Time qPCR, the GENETYX ver. 8.0 program, the BLASTX program from the National Center for Biotechnology Information (NCBI), the ProtParam tool from ExPASy Proteomics Serve, Simple Modular Architecture Research Too and the MEGA 4 program.*
Data format	*Analysed*
Experimental factors	*The molecular characteristics of the PmSCAMP5 gene were identified, and PmSCAMP5 gene expression level profiles were compared between healthy conditions and conditions after pathogenic challenges.*
Experimental features	*This experiment can provide information on PmSCAMP5 in the red sea bream, and the results should be used as basic data on for a functional analysis of PmSCAMP5.*
Data source location	*Gyeongsang National University, Tongyeong, Republic of Korea*
Data accessibility	*The data are available for this article*

**Table:** 

**Value of the data** • These data provide information on the molecular features of PmSCAMP5 in red sea bream and shows the level and patterns of PmSCAMP5 mRNA expression expected to be involved immune system. • These data provide a basis data for predicting the function of SCAMP5 through phylogenetic analysis of SCAMP5 and other species of SCAMP5. • These data will provide the baseline data for understanding the role of PmSCAMP5 in red sea bream infectedwith various pathogens.

## 1 Data

**Fig. 1:**
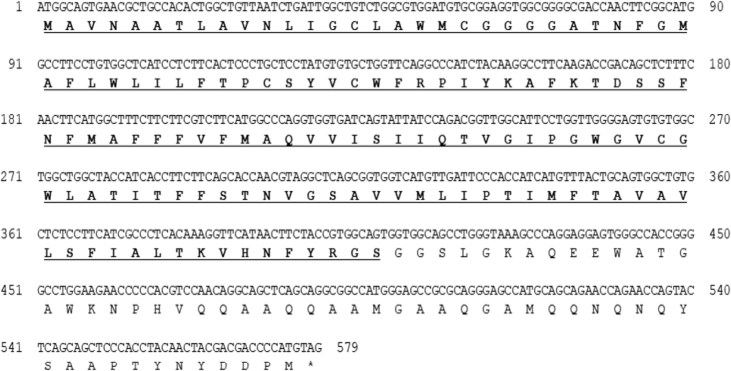
cDNA and the deduced amino acid sequence of PmSCAMP5. The PmSCAMP5 domain is underlined.

**Fig. 2:**
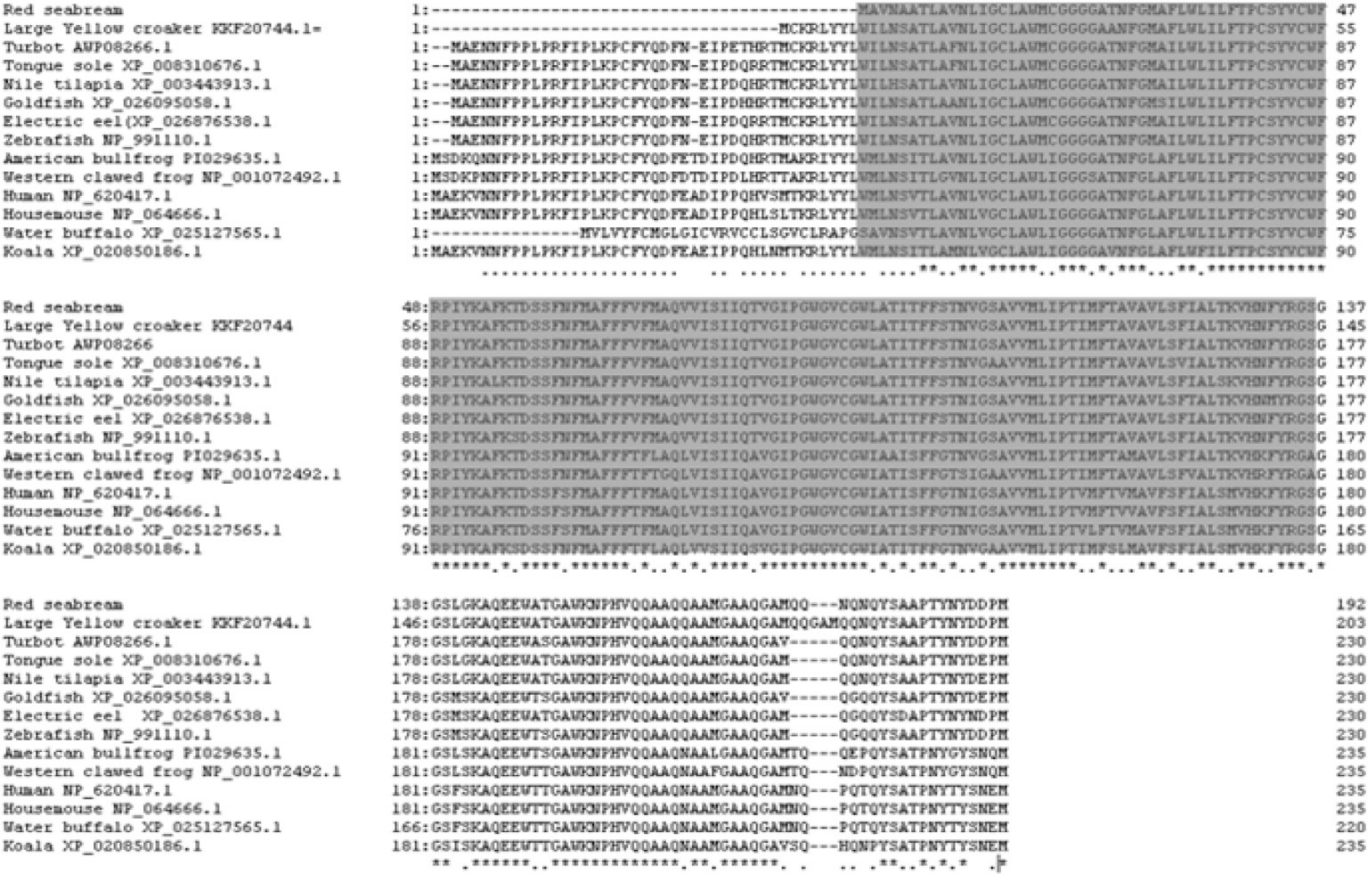
Multiple alignments of PmSCAMP5 with SCAMP5 amino acid sequences from other species. The NCBI accession numbers of dicentracin are as follows: Large yellow croaker, KKF20744.1; Turbot, AWP08266; Tongue sole, XP 008310676.1; Nile tilapia, XP 003443913.1; Goldfish, Xp 026095058.1; Electric eel, XP 026876538.1; Zebrafish, NP 991110.1; American bullfrog, PIO29635.1; Western clawed frog, NP 001072492.1; Human, NP 620417.1; Housemouse, NP 064666.1; Water buffalo, XP 025127565.1; Koala, XP 020850186.1.

**Fig. 3:**
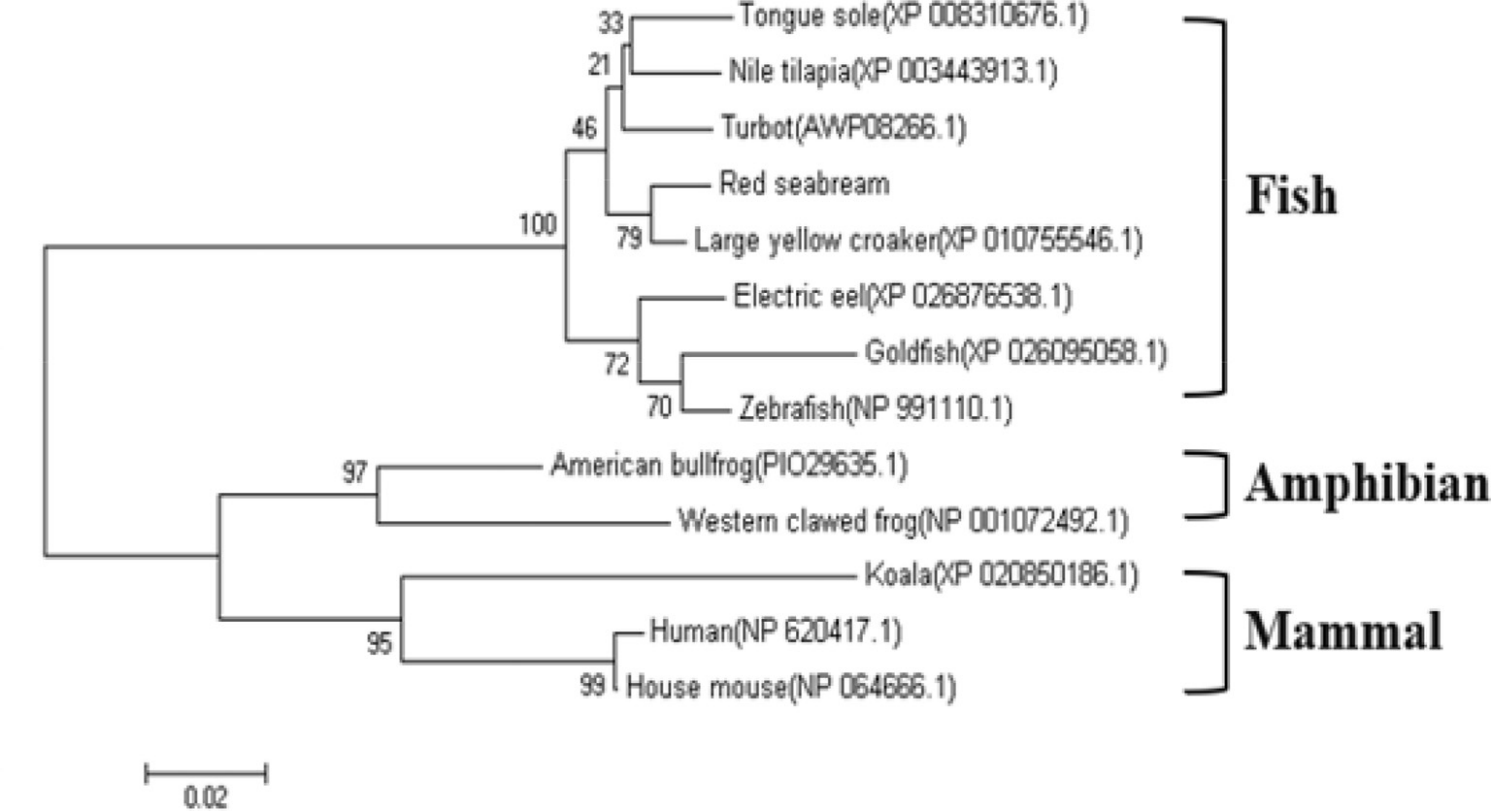
Phylogenetic analysis of the deduced SCAMP5 amino acid sequences in fish. The phylogenetic tree was constructed using the neighbour-joining method in MEGA 4 software. Bootstrap sampling was performed with 2000 replicates. The scale bar is equal to 0.02 changes per amino acid position.

**Fig. 4:**
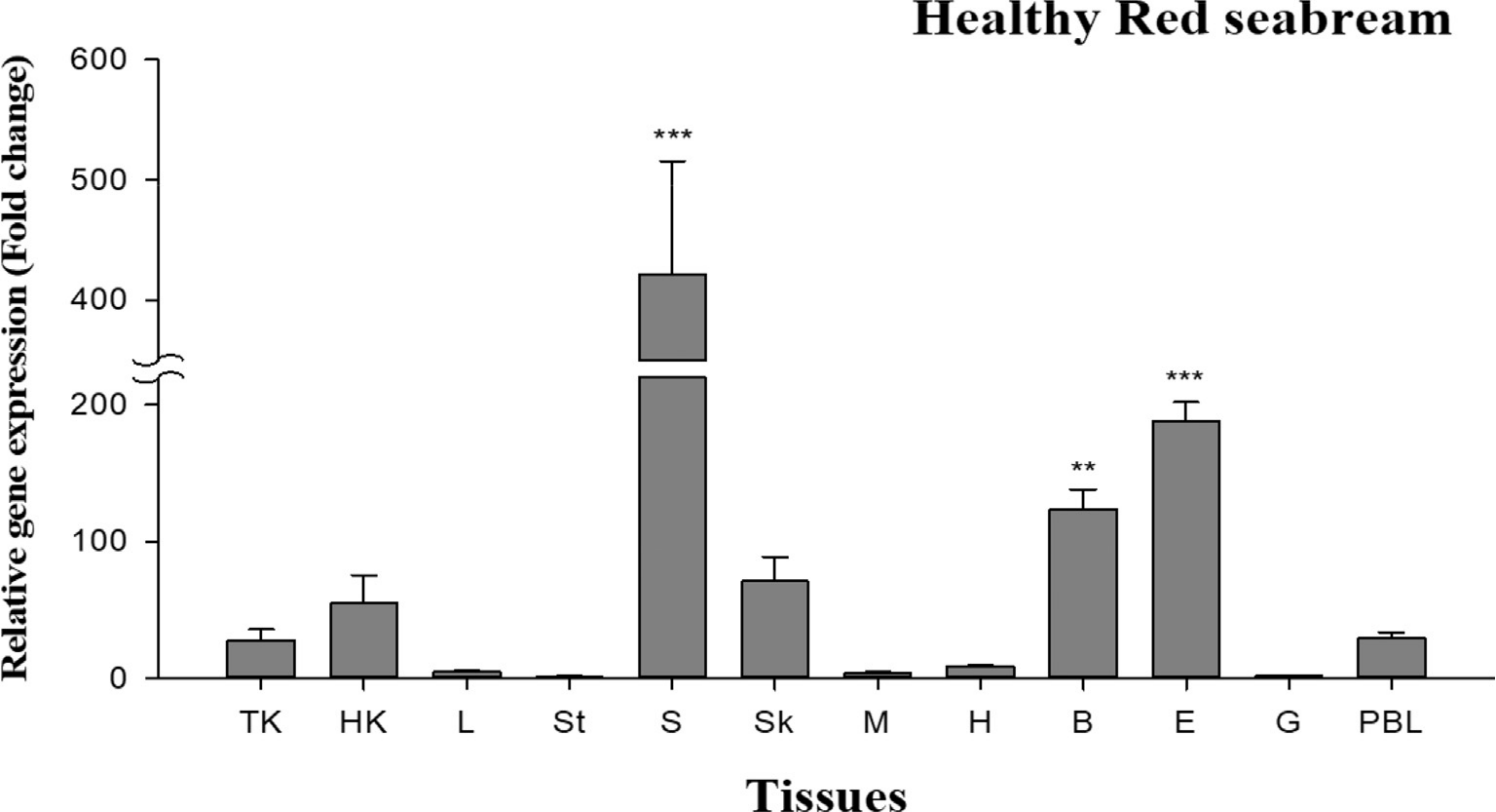
Detection of PmSCAMP5 mRNA expression in various tissues from healthy red seab ream by real-time PCR. EF-1α was used for normalizing the real-time PCR results. Data are presented as the mean ± SD from three independent cDNA samples with three replicates from each sample. The asterisks indicate significant differences (*P value < 0.05 and ** *P* value < 0.01) compared to the stomach.

**Fig. 5:**
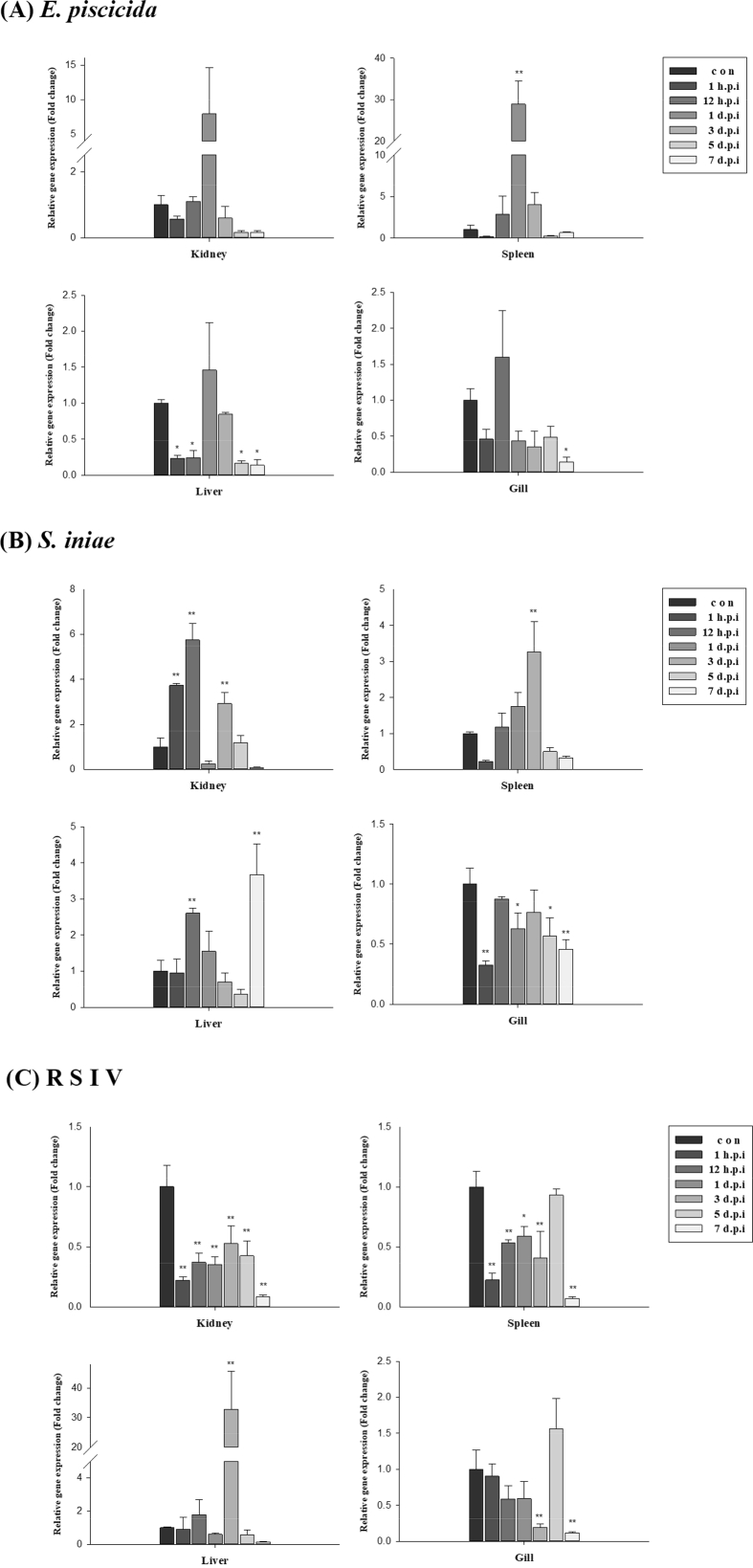
PmSCAMP5 mRNA expression levels in various tissues of red sea bream infected with three pathogens [(A) *Edwardsiella piscicida* (*E. piscicida*), (B) *Streptococcus iniae* (*S. iniae*) and (C) Red sea bream iridovirus (RSIV)]. The levels of PmSCAMP5 transcripts were quantified relative to the EF-1α levels. The data are presented as the mean ± SD from three independent cDNA samples with three replicates for each sample. The asterisks represent significant differences compared to the control (PBS) group from ANOVA (*P value < 0.05 and ** *P* value < 0.01).

The size of the SCAMP5 (PmSCAMP5) open reading frame (ORF) identified from the livers of red sea bream (*Pagrus major*) was 579 bp and 192 amino acids were encoded. An among them, there was an ORF domain of 136 amino acids. The PmSCAMP5 gene was predicted to have an isoelectric point (pI) of 8.30 and a molecular weight of 20.8 kDa (Fig. 1). PmSCAMP5 showed the highest homology compared to SCAMP5 of the Large yellow croaker. In contrast, SCAMP5 in zebrafish had a relatively low homology of 88% (Fig. 2). Analysis of the phylogenetic tree of the SCAMP5 gene divide clusters of mammals, amphibians and fishes, and SCAMP5 of red sea bream showed the closest relationship to SCAMP5 from the large yellow croaker (Fig. 3). Using quantitative real-time PCR (RT-qPCR), we evaluated PmSCAMP5 mRNA expression in healthy and pathogen challenged red sea bream. The PmSCAMP5 gene expression levels in healthy red sea bream were the higher in the spleen (421.37-fold), than the stomach (Fig. 4). In pathogen-challenged red sea bream, various expression patterns of PmSCAMP5 gene were observed in 4 tissues from red sea bream infected with pathogens (Fig. 5). After a challenge with *E. piscicida,* expression of PmSCAMP5 was significantly up-regulated in the spleen at 3 d.p.i, and PmSCAMP5 expression in other organs also showed a similar tendency to increase (Fig. 5A). For infection with the *S. iniae,* expression of PmSCAMP5 was significantly up-regulated in the kidneys at 1, 12 h.p.i and 3 d.p.i, and significantly up-regulated in the spleen at 3 d.p.i, and it was significantly up-regulated in the liver at 12 h.p.i and 7 d.p.i. In contrast, PmSCAMP5 expression levels in the gills tended to be significantly down-regulated (Fig. 5B). After challenge with the RSIV, expression of PmSCAMP5 was significantly down-regulated in the kidneys and spleens. However, the expression of PmSCAMP5 was significantly up-regulated in the liver after 3 days and the gills showed a pattern similar to the liver (Fig. 5C).

## 2 Experimental design, materials, and methods

### 2.1 Molecular characterization and phylogenetic analysis

An ORF sequence of the PmSCAMP5 gene was obtained from the liver of red sea bream stimulated with lipopolysaccharide (LPS) through Next Generation Sequencing (NGS) analysis. The amino acid sequence of PmSCAMP5 was predicted using the GENETYX ver. 8.0 program (SDC Software Development, Japan) and the BLASTX program of the National Center for Biotechnology Information (NCBI). The molecular weight and isoelectric point were predicted using the ProtParam tool of ExPASy Proteomics Serve, and specific domain was identified using Simple Modular Architecture Research Too. The multiple alignment of the PmSCAMP5 amino acid sequence and the SCAMP5 amino acid sequence of other species registered in peptide sequence database of NCBI was analysed using ClustalW analysis. The phylogenetic analysis of PmSCAMP5 was performed using the neighbour-joining (NJ) method of MEGA 4 program and bootstrap sampling was repeated 2,000 times [1].

### 2.2 Experimental animal and microbes

Healthy red sea bream (weight: 68.5 ± 10 g, body length: 14.3 ± 1 cm) without a disease history were provided by Gyeongsangnam-do Fisheries Resources Research Institute (Tongyeong, Republic of Korea), and they were acclimatized in a seawater tank (Water Temperature: 21.5 ± 1.5 °C) for 2 weeks before the experiment. In all experiments, the animals were anaesthetized with benzocaine (Sigma, St. Louis, MO) before tissue collection. For the bacterial and virus challenge experiment, *Edwardsiella piscicida (E. piscicida), Streptococcus iniae (S. iniae)* and Red sea bream iridovirus (RSIV) were obtained from the Fish Pathology Division of the National Institute of Fisheries Science (Busan, Republic of Korea).

### 2.3 Infection experiment

The red sea bream used in the experiment were divided into experimental and control groups. The experimental group was subdivided into the *S. iniae*, *E. piscicida* and RSIV groups, and had 30 animals. Infection experiments were performed by intraperitoneal injection of a pathogen suspension of *S. iniae* (1.5 × 10^5^ CFU/fish), *E. piscicida* (1.5 × 10^5^ CFU/fish) or RSIV (1 × 10^5^ copies/fish). The tissues (Kidney, spleen, liver and gill) of three randomly selected experimental fishes were extracted after 0 (con.), 1- and 12-h post-infection (hpi) and 1, 3, 5- and 7-days post-infection (dpi). In the control group, 12 tissues (trunk kidney, head kidney, liver, stomach, spleen, skin, muscle, heart, brain, eye, gills and blood) were extracted from three healthy red sea bream. Foe the blood, peripheral blood leukocytes were isolated using a percoll (Sigma, USA) gradient. All extracted tissues were aseptically collected, flash frozen in liquid nitrogen, and stored at −80 °C until they were used for total RNA extraction.

### 2.4 Total RNA extraction and reverse transcription

Total RNA was extracted from various red sea bream tissues using a TRIzol-based (RNAiso Plus) reagent (Takara) according to the manufacturer's instructions. The extracted total RNA samples were treated with DNase I (Takara, Japan) to remove the remaining genomic DNA. The concentration and purity of the total RNA samples were calculated from measurements obtained with a NanoVue spectrophotometer (GE Healthcare, UK). Total RNA was used for cDNA synthesis using a PrimeScript™ 1st strand cDNA Synthesis Kit (Takara) according to the manufacturer's instructions.

### 2.5 RT-qPCR analysis of PmSCAMP5 and statistical analysis

The synthesized cDNAs were used for a RT-qPCR analysis performed using gene-specific primer sets (SCAMP5 primer forward: 5′ -GGTTCAGGCCCATCTACAAG- 3′; revers: 5′ —CCAACCAGGAATGCCAAC- 3′) on a Thermal Cycler DICE Real-Time System using TB Green™ Premix Ex Taq™ (Takara). The primer set used in RT-qPCR was produced by Primer 3 v. 0.4.0 (http://bioinfo.ut.ee/primer3-0.4.0/). The relative mRNA expression level of PmSCAMP5 was calculated using the comparative threshold cycle method (2^−ΔΔCT^) with elongation factor-1α used as a control. For tissues from healthy red sea bream, relative expression levels were compared to other tissues based on the lowest expression in the stomach (Fig. 4). The results are reported as the mean ± standard deviation (SD) and were assessed using one-way analysis of variance (ANOVA) followed by Duncan's test (*P value < 0.05 and ** *P* value < 0.01) in SPSS software 19.0 (IBM, USA) [1].
